# Challenges in Managing Rebleeding After Endoscopic Hemostasis With Over-the-Scope Clip for Colonic Diverticular Bleeding: A Case Series

**DOI:** 10.7759/cureus.78641

**Published:** 2025-02-06

**Authors:** Takashi Nishino, Chikamasa Ichita, Chihiro Sumida, Miki Nagayama, Souichirou Nakaya, Akiko Sasaki

**Affiliations:** 1 Gastroenterology Medicine Center, Shonan Kamakura General Hospital, Kamakura, JPN

**Keywords:** acute lower gastrointestinal bleeding, colonic diverticular bleeding, colonic diverticular hemorrhage, endoscopic hemostasis, over-the-scope clips

## Abstract

Colonic diverticular bleeding is a leading cause of lower gastrointestinal bleeding, accounting for the increasing hospitalization rate in Japan. Although over-the-scope clips (OTSCs) have demonstrated favorable outcomes as an initial hemostatic method, their effectiveness as a rescue therapy after rebleeding from primary endoscopic hemostasis remains unclear. This study describes four patients with colonic diverticular bleeding who underwent an OTSC procedure after rebleeding from primary endoscopic hemostasis using standard methods. Immediate hemostasis was achieved in all cases; however, three patients experienced rebleeding during hospitalization, requiring interventional radiology or surgical intervention. Although immediate hemostasis can be achieved using OTSCs in cases of rebleeding after primary endoscopic hemostasis, their effectiveness in reducing short-term rebleeding might be limited. Further large-scale studies are warranted to evaluate OTSC efficacy in managing colonic diverticular bleeding after rebleeding from primary endoscopic hemostasis.

## Introduction

Colonic diverticular bleeding accounts for ≥60% of hospitalizations for lower gastrointestinal bleeding [1]. Particularly, the hospitalization rate for colonic diverticular bleeding in Japan has more than doubled, from 15.1 per 100,000 people in 2012 to 34.0 in 2019 [2]. The number of hospitalized patients with colonic diverticular bleeding surpassed that of patients with hemorrhagic gastric ulcers by 2017 [2], emphasizing the growing clinical significance of effective management of colonic diverticular bleeding. Unlike hemorrhagic gastric ulcers, which can be effectively treated or prevented by eradicating Helicobacter pylori and proton pump inhibitors, definitive pharmacological therapy for colonic diverticular bleeding remains lacking, with endoscopic hemostasis being a critical intervention.

Currently, standard endoscopic treatments include clipping and ligation methods, such as endoscopic band ligation (EBL), and the use of an over-the-scope clip (OTSC) for hemostasis has also been reported [3-5]. OTSC is a mechanical hemostatic device that allows for the compression of a larger amount of tissue compared to standard clips, making it particularly useful for cases where conventional clipping is ineffective [6]. Although the OTSC procedure, as an initial hemostatic method, exhibits relatively favorable outcomes [4,5], its efficacy in cases of primary endoscopic hemostasis failure remains unexplored.

This case series reports the use of OTSCs as a rescue therapy for colonic diverticular bleeding in patients who experienced rebleeding after initial standard endoscopic hemostasis.

## Case presentation

Case 1

**Figure 1:**
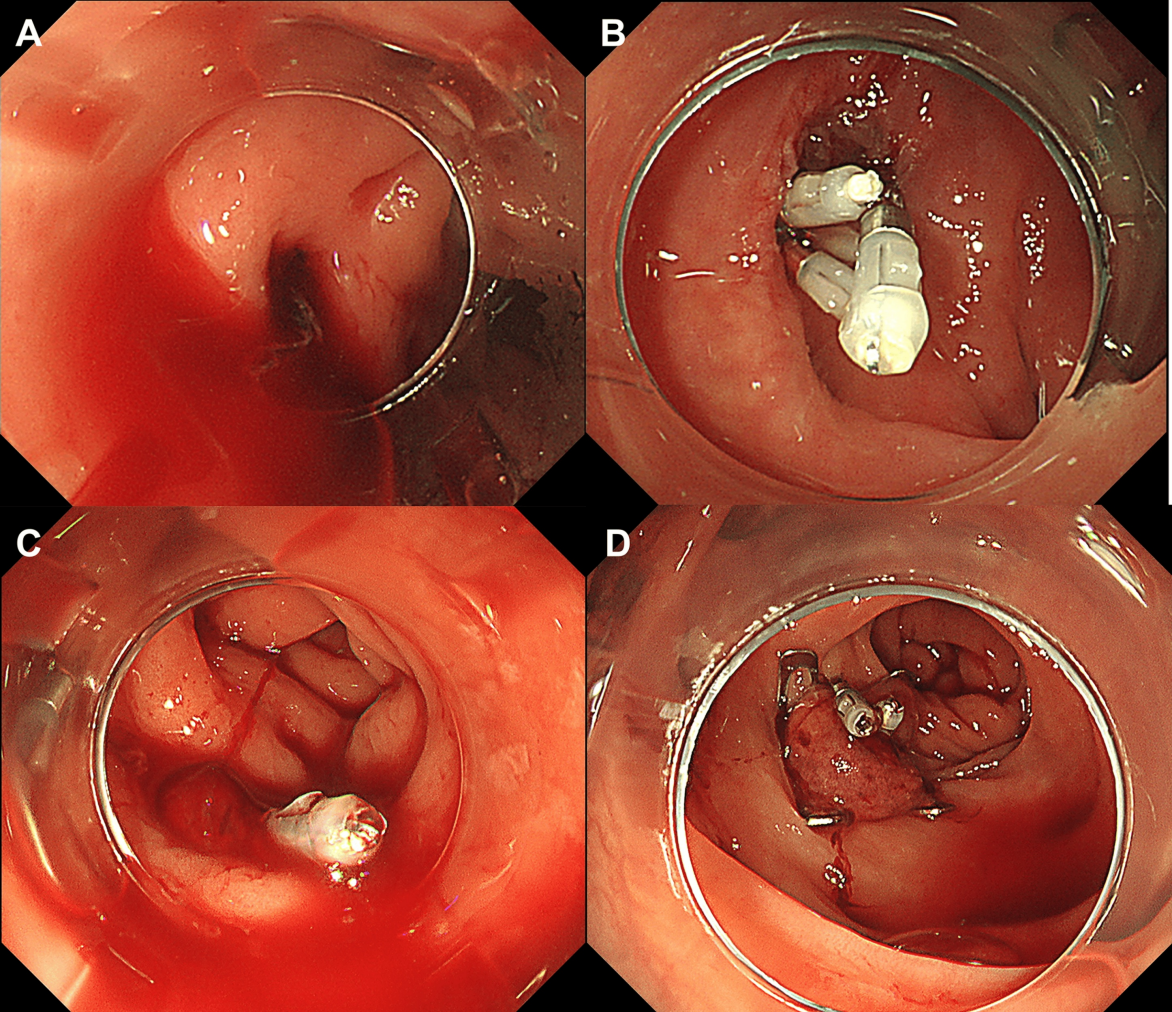
Endoscopic images from Case 1 A. Colonoscopy showing active bleeding from the ascending colon diverticulum B. Initial hemostasis achieved with direct clipping C. Rebleeding at the clipped site during repeat colonoscopy D. Hemostasis achieved using over-the-scope clip over residual clips

A 74-year-old man with no history of colonic diverticular bleeding presented with persistent hematochezia. The patient had no history of antithrombotic drug use, and his hemoglobin level was 11.7 g/dL on admission, with no signs of hypovolemic shock. The platelet count and coagulation parameters were within normal ranges. A colonoscopy performed 15 hours after arrival revealed active bleeding from the ascending colon diverticulum (Figure 1A). Initial hemostasis was achieved through direct clipping (Figure 1B). However, persistent hematochezia the following day necessitated repeated colonoscopy, revealing rebleeding at the clipped site (Figure 1C). Subsequently, an OTSC was applied over the residual clips to achieve hemostasis (Video 1, Figure 1D). Hematochezia recurred on day one post-OTSC, prompting transcatheter arterial embolization (TAE) for definitive hemostasis. The patient was discharged four days after TAE without further bleeding.

**Video 1:** Hemostasis with over-the-scope clip (OTSC) following rebleeding after primary direct clip hemostasis in ascending colonic diverticular bleeding (Case 1)

Case 2

**Figure 2:**
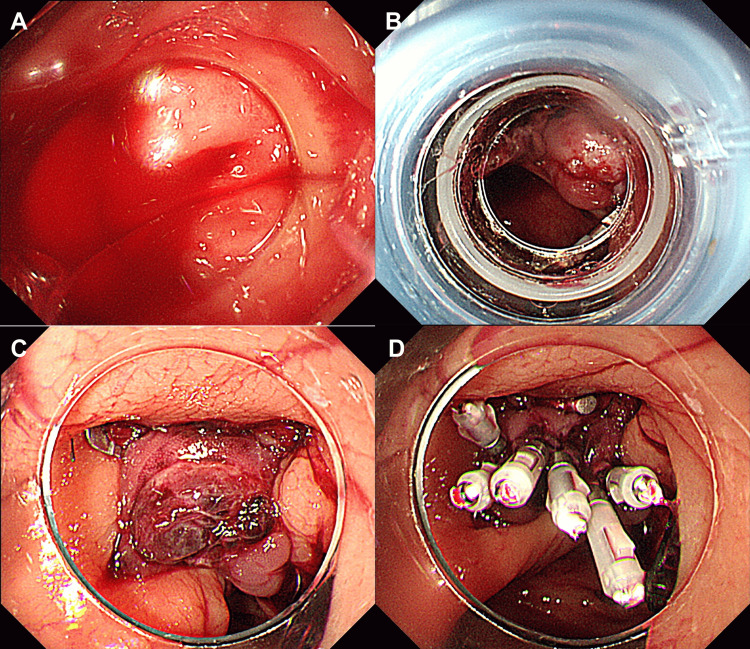
Endoscopic images from Case 2 A. Active bleeding from the ascending colon diverticulum near the cecum B. Successful hemostasis achieved with endoscopic band ligation C. Immediate hemostasis was achieved with an over-the-scope clip for rebleeding at the endoscopic band ligation site D. Hemostasis achieved using over-the-scope clips and additional clips for reinforcement

**Figure 3:**
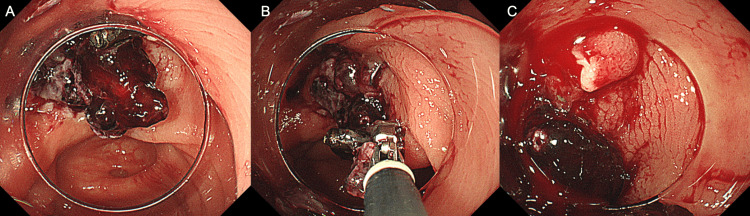
Confirmation of rebleeding from the over-the-scope clip (OTSC) site in Case 2 A. Colonoscopic image showing an adherent coagulum at the apex of the OTSC B. Image capturing the removal of the coagulum from the OTSC C. Persistent bleeding observed after coagulum removal

A 73-year-old man with a history of three episodes of colonic diverticular bleeding and aspirin use for angina presented with persistent hematochezia. His hemoglobin level was 12.5 g/dL on admission, and he exhibited hypotension with a systolic blood pressure of 80 mmHg. The platelet count and coagulation parameters were within normal ranges. Contrast-enhanced computed tomography (CT) revealed active extravasation in the ascending colon. A colonoscopy performed three hours after arrival revealed active bleeding from the ascending colon diverticulum near the cecum (Figure 2A). Subsequently, EBL was successfully performed (Figure 2B). Hematochezia recurred on day one post-EBL. Repeat colonoscopy revealed persistent bleeding at the EBL site with adherent coagulum. The OTSC was applied to successfully achieve hemostasis (Figure 2C). Clips were also applied over the ulcer base on top of OTSC to prevent recurrent bleeding (Figure 2D). Hematochezia recurred on day five post-OTSC. Colonoscopy revealed an adherent coagulum at the apex of the OTSC (Figure 3A). Upon removal of the coagulum (Figure 3B), persistent bleeding was observed (Figure 3C). Although temporary hemostasis was achieved with clipping, rebleeding occurred the following day, necessitating surgical intervention. The patient was discharged on postoperative day seven.

Case 3

**Figure 4:**
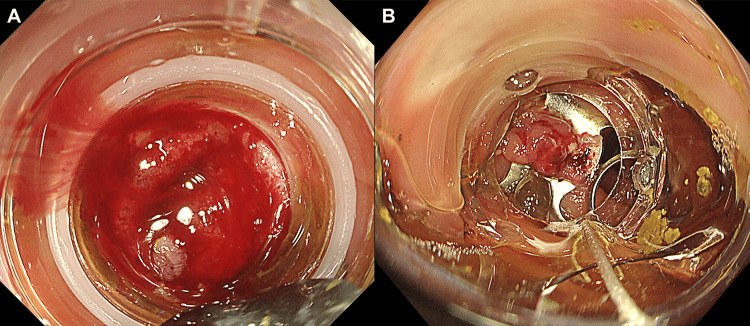
Endoscopic images from Case 3 A. Failure of initial hemostasis with endoscopic band ligation due to insufficient inversion in an ascending colon diverticulum B. Hemostasis achieved using over-the-scope clips

A 75-year-old man with a history of colonic diverticular bleeding one year ago and aspirin use for prior myocardial infarction presented with persistent hematochezia. His hemoglobin level was 13.9 g/dL on admission, with no signs of hypovolemic shock. The platelet count and coagulation parameters were within normal ranges. Contrast-enhanced CT revealed extravasation in the ascending colon. The initial colonoscopy revealed exposed vessels in the ascending colonic diverticulum. Notably, EBL failed due to the rigidity of the bowel wall and insufficient inversion (Figure 4A). Consequently, indirect clipping was performed to achieve hemostasis. Hematochezia recurred on day one post-clipping. OTSC was used to achieve hemostasis (Figure 4B). However, recurrent bleeding occurred on day one post-OTSC. Considering that the patient had undergone percutaneous coronary intervention for myocardial infarction three months earlier and exhibited a hemoglobin level decreased to 8.7 g/dL due to repeated bleeding, a right hemicolectomy was performed. The patient was discharged on postoperative day eight.

Case 4

**Figure 5:**
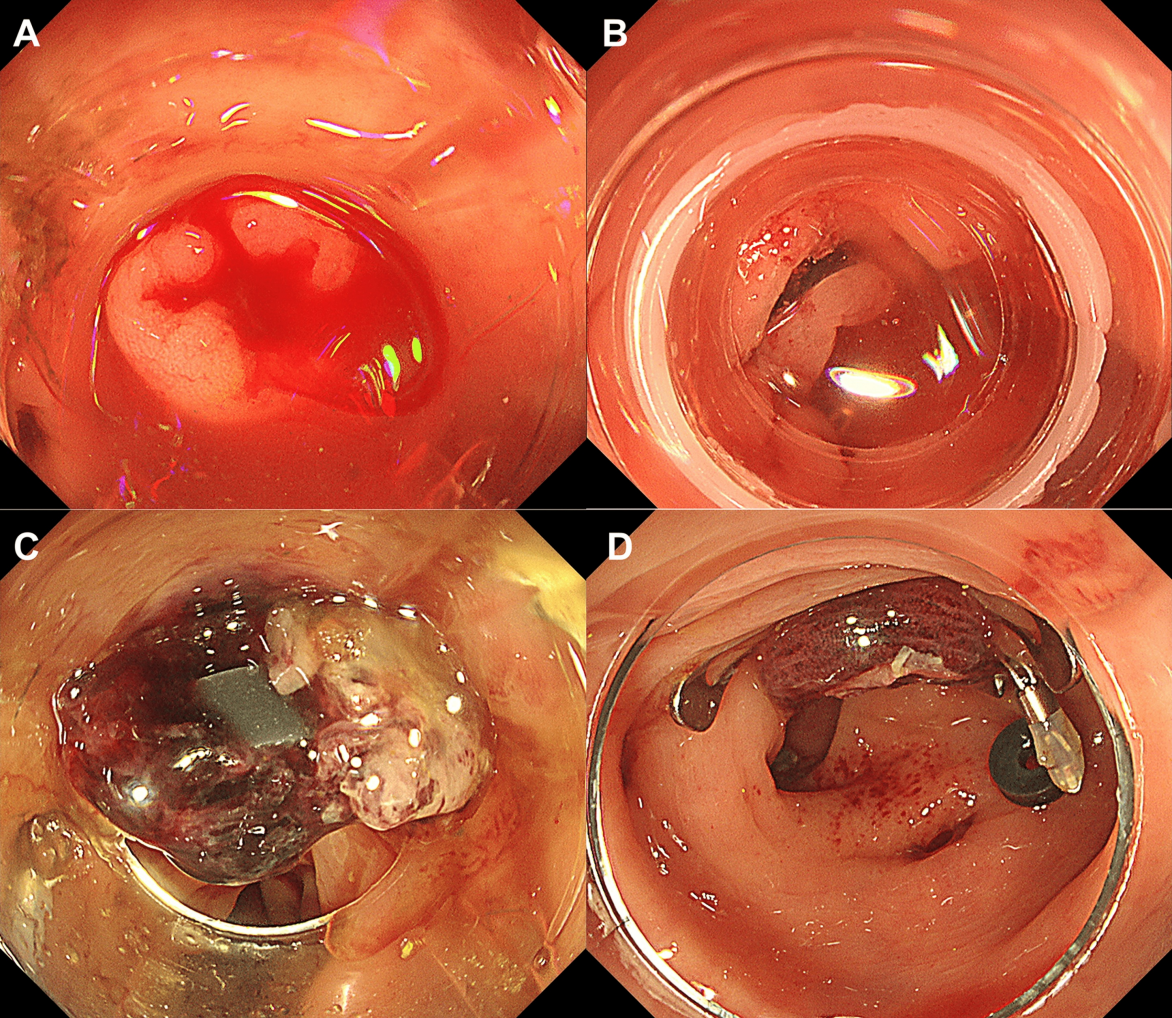
Endoscopic images from Case 4 A. Active bleeding from a sigmoid colon diverticulum B. Initial hemostasis achieved with endoscopic band ligation C. Mucosal fragility and adherent coagulum were observed at the endoscopic band ligation site, and rebleeding from the same site was confirmed D. Hemostasis achieved using over-the-scope clips

An 84-year-old man with 10 prior episodes of colonic diverticular bleeding and aspirin use for angina presented with persistent hematochezia. His hemoglobin level was 10.4 g/dL, on admission, with no signs of hypovolemic shock. The platelet count and coagulation parameters were within normal ranges. A colonoscopy performed 49 hours after admission revealed active bleeding from the sigmoid colonic diverticulum (Figure 5A). Subsequently, EBL was successfully performed (Figure 5B). Hematochezia recurred on day two post-EBL. A repeat colonoscopy revealed persistent bleeding at the EBL site with an adherent coagulum (Figure 5C). OTSC was used to achieve hemostasis (Figure 5D). The patient was discharged on post-OTSC day five. This case experienced rebleeding six months after discharge; however, the bleeding originated from a different diverticulum rather than the lesion treated with OTSC.

## Discussion

The details of this case series are summarized in Table 1. This case series demonstrates the use of OTSC as a rescue therapy for colonic diverticular bleeding in patients who experienced rebleeding after standard endoscopic hemostasis. Immediate hemostasis was successfully achieved using OTSC in all four patients. However, three patients subsequently experienced rebleeding during hospitalization, necessitating TAE or surgical intervention.

**Table 1:** Summary of the case series OTSC, Over-The-Scope Clip; TAE, transcatheter arterial embolization, EBL, endoscopic band ligation

Case	Age	Sex	Bleeding site	Initial hemostasis	Antithrombotic agents use	Days to rebleeding after initial hemostasis	Immediate hemostasis with OTSC	In-hospital rebleeding after OTSC	Days to rebleeding post-OTSC	Additional Treatment	Length of hospital stay
1	74	Male	Ascending	Direct clipping	No	1	Yes	Yes	1	TAE	10
2	73	Male	Ascending	EBL	Aspirin	1	Yes	Yes	5	Surgery	15
3	75	Male	Ascending	Indirect clipping	Aspirin	1	Yes	Yes	1	Surgery	14
4	84	Male	Sigmoid	EBL	Aspirin	2	Yes	No	-	-	11

Systematic reviews have demonstrated the utility of OTSC for gastrointestinal bleeding [7-9]. However, only two case series are available for colonic diverticular bleeding, reporting short-term rebleeding rates of 33.3% (2/6) and 8.3% (3/36) after the OTSC procedure [4,5], indicating relatively favorable outcomes. Meanwhile, the largest existing studies on standard hemostatic methods have reported varying short-term rebleeding rates of 18.6% (67/360), 27.8% (189/681), and 13.2% (84/638) for the direct clipping method, indirect clipping method, and EBL, respectively [10,11]. When initial hemostasis fails, a clear bleeding point is often not identifiable, leaving indirect clipping - the hemostatic method with the highest rebleeding rate - as the only available option. Given this limitation, OTSC was considered a potentially effective alternative for rescue hemostasis. The "jump effect" of OTSC, wherein the clip advances approximately 4 mm upon closure [5], allows hemostasis even in challenging situations, such as the presence of residual clips following initial clipping or O-ring detachment after EBL [12]. As demonstrated in our Video 1 and Figure 1D, OTSC application may achieve hemostasis even when the bleeding point cannot be clearly identified or suction is inadequate. Beyond hemostasis, OTSC's strong gripping force may also help prevent delayed perforation, providing an additional potential benefit. However, the results of our study demonstrated that OTSC as a rescue method did not achieve favorable outcomes, with an in-hospital rebleeding rate of 75%.

Several factors may explain the higher short-term rebleeding rate when OTSC was used as a rescue therapy rather than for primary hemostasis. In some cases, such as Case 3, fibrosis around the responsible diverticulum after the initial hemostasis attempt may have made suction difficult, reducing the effectiveness of OTSC despite the jump effect. Additionally, a high blood flow into the responsible diverticulum may have exceeded the hemostatic capacity of OTSC. The design of available OTSC types (t-type and gc-type) in Japan includes gaps between their "claws," which may allow blood perfusion through tissue, leading to temporary hemostasis followed by recurrent bleeding. This mechanism has been observed in post-endoscopic submucosal dissection duodenal ulcer cases and may have contributed to rebleeding in our study [13], particularly in Case 2, where additional preventive clips placed over the OTSC did not successfully prevent rebleeding. Moreover, in two out of three cases of failed OTSC rescue, patients were taking aspirin. The use of antithrombotic agents, including aspirin, may have contributed to the higher short-term rebleeding rate. Furthermore, in cases following EBL, ischemic changes likely occurred, leading to tissue fragility and delayed tissue healing, which may have increased the risk of rebleeding. While these factors may have influenced the rebleeding rate, further studies with larger case series are needed to clarify the mechanisms underlying the higher rebleeding rate of OTSC as a rescue therapy.

OTSC has two major limitations: cost and technical challenges. The cost of OTSC is ¥79,800 (excluding tax) (532 USD, based on an exchange rate of 150 JPY/USD), whereas EBL costs ¥10,000 (excluding tax) (67 USD), and clips range from ¥1,000 to ¥2,000 (excluding tax) (approximately 7-13 USD) per unit, depending on the type. OTSC is approximately eight times more expensive than other devices. From a technical standpoint, OTSC placement requires complete scope removal followed by reinsertion with a relatively large device attached. Some patients with colonic diverticular bleeding have a narrowed lumen due to multiple diverticula, which can make reinsertion challenging. While this was not an issue in our cases, such anatomical constraints could pose difficulties in certain clinical scenarios. Given these economic and technical limitations, the indication for OTSC should be carefully considered in clinical practice.

## Conclusions

In our study, the short-term rebleeding rate of OTSC as a rescue therapy following rebleeding after primary standard hemostasis was high at 75%. Considering the cost implications, its routine use for all cases cannot be recommended at this stage. However, given the small sample size of this case series, further large-scale investigations are indeed warranted to evaluate its efficacy and establish criteria for its appropriate application.
